# Clinical Significances of Positive Postoperative Serum CEA and Post-preoperative CEA Increment in Stage II and III Colorectal Cancer: A Multicenter Retrospective Study

**DOI:** 10.3389/fonc.2020.00671

**Published:** 2020-05-20

**Authors:** Weiqiang You, Li Yan, Zerong Cai, Lei Xie, Nengquan Sheng, Guiyu Wang, Xiaojian Wu, Zhigang Wang

**Affiliations:** ^1^Department of General Surgery, Shanghai Jiao Tong University Affiliated Sixth People's Hospital, Shanghai, China; ^2^Guangdong Provincial Key Laboratory of Colorectal and Pelvic Floor Diseases, Department of Colorectal Surgery, Guangdong Institute of Gastroenterology, The Sixth Affiliated Hospital, Sun Yat-sen University, Guangzhou, China; ^3^Department of General Surgery, The Second Affiliated Hospital of Harbin Medical University, Harbin, China

**Keywords:** colorectal cancer, carcinoembryonic antigen, prognosis, stage II, stage III

## Abstract

**Background:** Carcinoembryonic antigen (CEA) is the most common serum tumor marker in colorectal cancer (CRC). Nevertheless, few previous studies demonstrated the impacts of postoperative CEA and post-preoperative CEA increment on prognosis of CRC.

**Methods:** Patients with stage II and III CRC were included from January 2009 to December 2015. All clinical and follow-up data were collected. Patients were divided into four different groups according to the levels of postoperative serum CEA and post-preoperative CEA trends. Chi-square test was used to analyze the relationship between clinical variables and categorized postoperative CEA and CEA increment. Cox proportional hazard regression was used for univariate and multivariable analyses. The log-rank test was performed to compare PFS and OS among groups.

**Results:** Patients, 1,008, who underwent radical surgery, were enrolled. Our results showed that positive postoperative CEA and CEA increment were related to clinical stage, T stage, N stage, tumor differentiation, and lymphatic invasion (*p* < 0.05). Univariate and multivariable analysis results suggested that positive postoperative CEA and CEA increment were independent prognostic factors for PFS (HR = 3.149, 95% CI, 2.426–4.088, *p* = 0.000 for postoperative CEA; HR = 2.708, 95% CI, 2.106–3.482, *p* = 0.000 for CEA increment) and OS (HR = 3.414, 95% CI, 2.549–4.574, *p* = 0.000 for postoperative CEA; HR = 2.373, 95% CI, 1.783–3.157, *p* = 0.000 for CEA increment). The survival analyses revealed positive postoperative CEA, and CEA increment predicted worse prognosis. Furthermore, our results indicated that the 3- and 5-year PFS rates were 86.6 and 78.4% in group A, but decreased to 25.3 and 7.2% in group D (*p* < 0.001). Similarly, the 3- and 5-year OS rates for group A were 92.5 and 83.9%, much higher than group D (*p* < 0.001). In other words, patients with both postoperative CEA elevation and CEA increment had the worst prognosis.

**Conclusions:** Positive postoperative CEA and CEA increment were independent prognostic factors for stage II and III CRC. Additionally, postoperative CEA and CEA increment had significant impacts on PFS and OS of CRC.

## Introduction

Colorectal cancer (CRC) is one of the most commonly diagnosed cancers worldwide with high morbidity and mortality rates (1). In recent years, although the treatment of colorectal cancer has been greatly developed, 5-year survival rate is only 67% for patients with rectal cancer, slightly higher than 64% with colon cancer (2). In China, incidence rate of CRC has been increasing year by year from 2000 to 2011 due to westernization of lifestyle (3).

The carcinoembryonic antigen (CEA) is mainly secreted by solid tumors. In CRC, CEA has always been recommended as a reliable tumor marker by the National Comprehensive Cancer Network (NCCN) and the American Society of Clinical Oncology (4). CEA plays an important role in diagnosis, postoperative recurrence, and metastasis, and the effect of chemotherapy of CRC (5–7). High levels of preoperative serum CEA always indicate worse prognosis and shorter progression-free survival time (PFS) in CRC (8, 9). Besides, postoperative CEA level is an independent prognosis index for CRC and its positivity reflects the probability of liver metastasis after surgery (10, 11). Increased postoperative CEA level at short intervals indicates the possibility of CRC recurrence and suggests that patients should be followed up more frequently (12). For metastasis CRC (mCRC), baseline level of CEA predicts the efficacy of some chemotherapy drugs and provides different information of overall survival time (13, 14). Several studies have also elucidated the effect of post/preoperative CEA ratio on the treatment of CRC, and post/preoperative CEA ratio <1 reveals a better prognosis than CEA ratio >1 for CRC (15, 16).

Generally speaking, the value of CEA in prognosis of CRC has well been demonstrated. However, few studies have systematically analyzed the significances of postoperative CEA level and post-preoperative CEA increment for the prognosis of stage II and III CRC after radical resection. Therefore, we conducted this multicenter retrospective clinical trial to analyze the importance of postoperative CEA and post-preoperative CEA increment in survival of CRC patients.

## Methods

### Data Collection

This study was a multicenter retrospective clinical study, and it was registered in the Chinese Clinical Trial Registry (Approval No. ChiCTR1800016906). Our study was also approved by the ethics committee of Shanghai Jiao Tong University Affiliated Sixth People's Hospital (Approval No. 2018-KY-031K). All of the patients are from Shanghai Jiao Tong University Affiliated Sixth People's Hospital, the Second Affiliated Hospital of Harbin Medical University, and the Sixth Affiliated Hospital of Sun Yat-sen University. All patients were pathologically diagnosed as stage II and III CRC from January 2009 to December 2015. Written informed consents were obtained from all patients in this study. The criteria for exclusion were as follows: (1) without available postoperative CEA value within 12 weeks after surgery; (2) loss to follow-up; (3) unsuitable pathological type; (4) without available preoperative CEA value.

The clinical characteristics of the patients, including gender, age, CEA value, and pathological reports, were all acquired from electronic patients' records and the departmental database. Pathological reports are detailed description of the surgical excised colorectal tissues, including T stage, N stage, pathological type, tumor differentiation, lymphatic invasion, and vascular invasion. Pathological stage was defined according to the 8th AJCC criterion for CRC. T stage meant the depth of primary tumor infiltration and N stage represented the number and extent of lymph node metastasis. Preoperative CEA value was tested within 1 week before surgery, and postoperative CEA value was gained within 12 weeks after surgery but before medical treatment. The value of CEA > 5 ng/ml is defined as positive (17). Patients were grouped as follows: (Group A) normal postoperative CEA (≤ 5 ng/ml) and without post-preoperative CEA increment; (Group B) normal postoperative CEA and with CEA increment; (Group C) positive postoperative CEA (>5 ng/ml) and without CEA increment; (Group D) positive postoperative CEA and with CEA increment. All patients were followed up according to current guidelines, including serum tumor markers, colonoscopy, chest X-ray, and CT (or MRI). Survival status and recurrence/metastasis status were updated by telephone, email, and medical history. Progression-free survival (PFS) was defined as the time from surgery to cancer metastasis or recurrence. Overall survival (OS) was defined as the time from surgery to death.

### Statistical Analysis

All data in this study were analyzed by IBM SPSS STATISTICS 22.0 software and GraphPad Prism 6. Categorical variables were compared using Pearson chi-square test. Survival rates, 3 and 5 years, were assessed by the Kaplan–Meier method, and the log-rank test was used to compare the differences in survival rates among different groups. Cox regression was used to test the effect of various indicators on the prognosis of CRC and estimate hazard ratios (HRs) and 95% CIs. *P*-values were two-sided, with statistically significant differences at *P* < 0.05.

## Results

### Patients' Information

**Figure 1:**
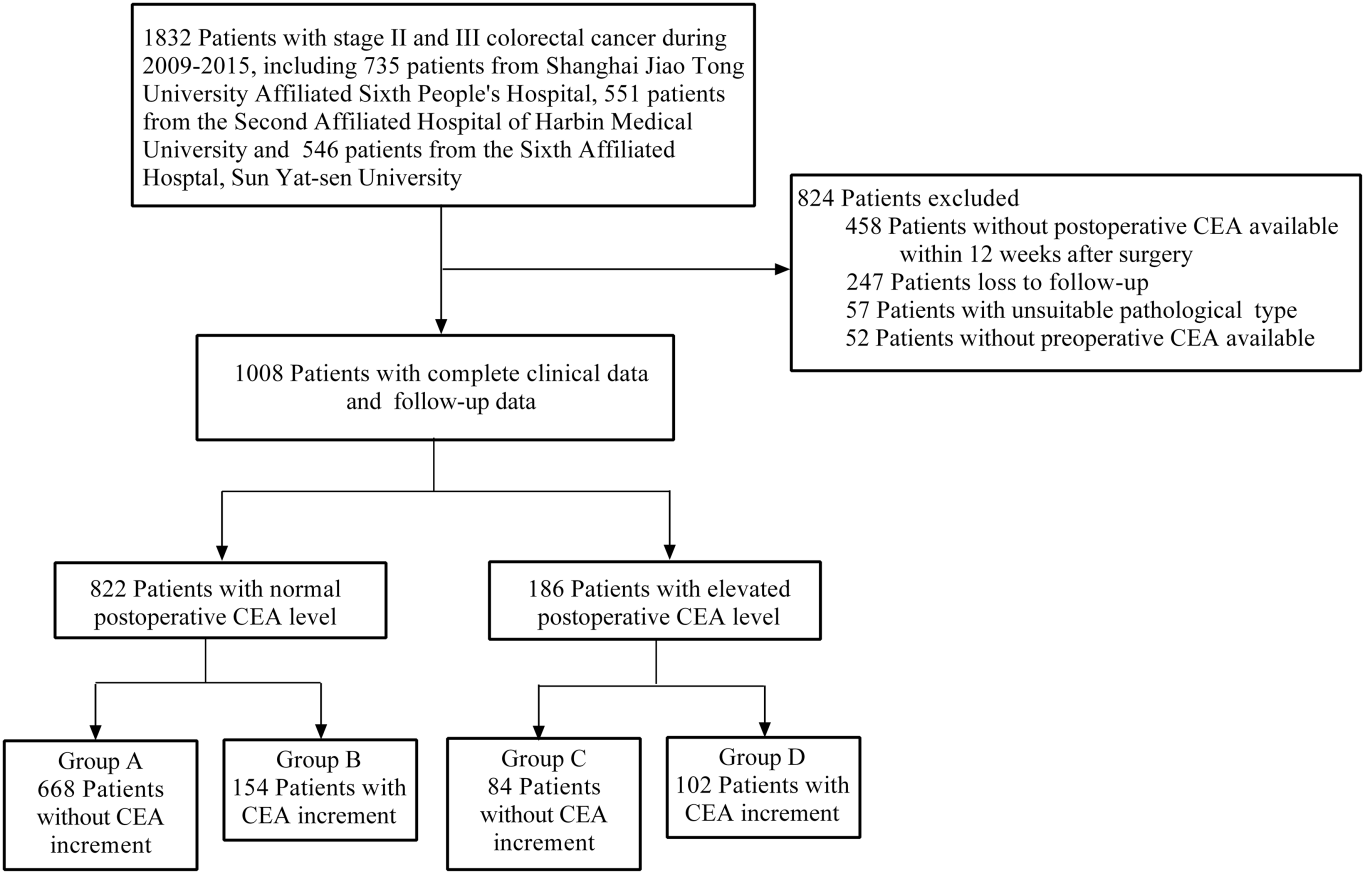
Study design.

A total of 1,832 patients with stage II and III CRC were enrolled in our study. According to our inclusion and exclusion criteria, 1,008 patients with complete clinical and follow-up data eventually were included. Furthermore, based on our grouped rules, the final number of patients in each group was as follows: (A) 668 patients; (B) 154 patients; (C) 84 patients; (D) 102 patients (Figure 1).

Our study included 605 males (60.0%) and 403 females (40.0%). The age of 671 patients (66.6%) was over 60. Patients, 573 (56.8%), were diagnosed as stage II CRC, slightly more than stage III CRC. Specifically, 546 patients (54.1%) had T3 stage, and 423 patients (42.0%) had T4 stage, while there were only 39 patients with T1 and T2 (3.9%). Patients, 435, had lymph node metastasis, including 280 cases (27.8%) with N1, and 155 cases (15.4%) with N2. The pathological type of 950 patients (94.2%) was adenocarcinoma. CRC tissues, 794 (78.8%), were well or moderately differentiated, while 214 tissues (21.2%) were poorly differentiated. According to our data, lymphatic invasion was observed in 298 patients (29.6%), and vascular invasion was found in 170 patients (16.9%). Only 186 patients (18.5%) had elevated postoperative CEA value even after radical surgery. Post-preoperative CEA increment was found in 256 patients (25.4%). The median follow-up time was 46 months. According to our follow-up data, 292 patients (29.0%) had recurrence or metastasis, and 224 patients (23.0%) died (Table 1).

**Table 1:** Patient demographics and clinicopathologic data.

**Variables**	**Patients (*N* = 1,008)**
**Gender**	
Male	605 (60.0%)
Female	403 (40.0%)
**Age**	
<60	337 (33.4%)
≥60	671 (66.6%)
**Clinical stage**	
II	573 (56.8%)
III	435 (43.2%)
**T stage**	
T1 + T2	39 (3.9%)
T3	546 (54.1%)
T4	423 (42.0%)
**N stage**	
N0	573 (56.8%)
N1	280 (27.8%)
N2	155 (15.4%)
**Pathological type**	
Adenocarcinoma	950 (94.2%)
Mucinous adenocarcinoma and signet ring cell carcinoma	58 (5.8%)
**Degree of differentiation**	
Moderate and well	794 (78.8%)
Poor	214 (21.2%)
**Lymphatic invasion**	
Positive	298 (29.6%)
Negative	710 (70.4%)
**Vascular invasion**	
Positive	170 (16.9%)
Negative	838 (83.1%)
**Postoperative CEA level**	
Positive (>5 ng/ml)	186 (18.5%)
Negative (≤ 5 ng/ml)	822 (81.5%)
**Post-preoperative CEA increment**	
Yes	256 (25.4%)
No	752 (74.6%)
**Metastasis or recurrence**	
Yes	292 (29.0%)
No	716 (71.0%)
**Survival status**	
Alive	776 (77.0%)
Dead	224 (23.0%)

### Positive Postoperative CEA and Post-preoperative CEA Increment Were Associated With Several Clinicopathologic Characteristics

To investigate the correlation of postoperative CEA level and CEA increment with clinical and pathological parameters, we did the chi-square tests to analyze. Our results showed that positive postoperative CEA was related to clinical stage, T stage, N stage, tumor differentiation, lymphatic and vascular invasion (all values of *p* < 0.05) (Table 2). In contrast, there was no significant difference in gender, age, or pathological type (Table 2). Besides, our data also found that post-preoperative CEA increment was significantly different in terms of clinical stage, T stage, N stage, tumor differentiation, and lymphatic invasion (all values of *p* < 0.05) (Table 2). However, there was no significant difference in terms of gender, age, pathological type, and vascular invasion (Table 2). In addition, the high levels of postoperative CEA and CEA increment suggested recurrence or metastasis and poor prognosis of CRC (*p* < 0.05) (Table 2).

**Table 2:** The association of clinicopathologic characteristics with postoperative carcinoembryonic antigen (CEA) level and post-preoperative CEA increment.

**Variables**	**Postoperative CEA**			**Post-preoperative CEA increment**		
	**Positive (*N* = 186)**	**Negative (*N* = 822)**	**p-Value**	**Yes (*N* = 256)**	**No (*N* = 752)**	**p-Value**
Gender			0.952			0.521
Male	112	493		158	447	
Female	74	329		98	305	
Age			0.471			0.691
<60	58	279		83	254	
≥60	128	543		173	498	
Clinical stage			0.004			0.007
II	88	485		127	446	
III	98	337		129	306	
T stage			0.000			0.000
T1 + T2	5	34		19	20	
T3	71	475		118	428	
T4	110	313		119	304	
N stage			0.006			0.013
N0	88	485		127	446	
N1	58	222		78	202	
N2	40	115		51	104	
Pathological type			0.423			0.310
Adenocarcinoma	173	777		238	712	
Other	13	45		18	40	
Differentiation			0.000			0.000
Moderate and well	122	672		176	618	
Poor	65	150		80	134	
Lymphatic invasion			0.000			0.000
Positive	82	216		98	200	
Negative	104	606		158	552	
Vascular invasion			0.000			0.130
Positive	49	121		51	119	
Negative	137	701		205	633	
Metastasis and recurrence			0.000			0.000
Yes	119	173		138	154	
No	67	649		118	598	
Survival status			0.000			0.000
Alive	81	695		145	631	
Dead	105	127		111	121	

### Univariate and Multivariable Analyses Revealed That Positive Postoperative CEA and Post-preoperative CEA Increment Were Independent Prognostic Factors for CRC

To examine the relationship between clinical variables and progression-free survival (PFS) and overall survival (OS) in CRC patients, we performed univariate and multivariable Cox regression analyses. Thirty-nine patients with T1 and T2 in our study were all stage III CRC with lymph node metastasis and had poor prognosis, which would have a large bias in univariate and multivariable analyses. Thus, these patients were excluded in this part. Our univariate analysis results suggested that clinical stage, T stage, N stage, tumor differentiation, lymphatic invasion, vascular invasion, postoperative CEA level, and CEA increment were prognostic factors of PFS and OS (all values of *p* < 0.05) (Table 3). However, gender, age, and center had no significance for PFS and OS. Furthermore, multivariable analysis results indicated that positive postoperative CEA was an independent prognostic factor for PFS (HR = 3.149, 95% CI, 2.426–4.088, *p* = 0.000) (Table 3) and OS (HR = 3.414, 95% CI, 2.549–4.574, *p* = 0.000) (Table 3). Similarly, our results also found that CEA increment had significant impacts on PFS (HR = 2.708, 95% CI, 2.106–3.482, *p* = 0.000) (Table 3) and OS (HR = 2.373, 95% CI, 1.783–3.157, *p* = 0.000) (Table 3). In general, our results demonstrated that positive postoperative CEA and CEA increment had great significances to the prognosis of patients with stage II and III CRC.

**Table 3:** Univariate and multivariable Cox regression analyses for progression-free survival time (PFS) and overall survival (OS) of colorectal cancer (CRC) patients (T1 and T2 were excluded).

**Variables**	**PFS**		**OS**	
	**HR (95% CI)**	***p*-Value**	**HR (95% CI)**	***p*-Value**
**Univariate analysis**				
Gender (male vs. female)	1.109 (0.874–1.408)	0.393	1.028 (0.789–1.339)	0.837
Age (<60 vs. ≥60)	1.093 (0.854–1.398)	0.479	1.209 (0.915–1.598)	0.183
Clinical stage (III vs. II)	2.350 (1.860–2.970)	0.000	2.965 (2.266–3.880)	0.000
T stage (T4 vs. T3)	1.418 (1.125–1.786)	0.003	1.812 (1.396–2.353)	0.000
N stage		0.000		0.000
N0	1 (Referent)		1 (Referent)	
N1	1.968 (1.498–2.585)	0.000	2.400 (1.760–3.274)	0.000
N2	3.033 (2.280–4.033)	0.000	3.987 (2.902–5.477)	0.000
Pathology type (adenocarcinoma vs. other)	0.692 (0.452–1.059)	0.090	0.577 (0.372–0.895)	0.014
Differentiation (moderate and well vs. poor)	2.014 (1.573–2.579)	0.000	2.267 (1.728–2.975)	0.000
Lymphatic invasion (positive vs. negative)	1.881 (1.487–2.379)	0.000	2.620 (2.021–3.395)	0.000
Vascular invasion (positive vs. negative)	1.397 (1.057–1.846)	0.019	1.966 (1.478–2.615)	0.000
Postoperative CEA level (positive vs. negative)	4.620 (3.646–5.854)	0.000	5.196 (4.001–6.748)	0.000
CEA increment (yes vs. no)	3.822 (3.031–4.820)	0.000	3.715 (2.867–4.812)	0.000
Centers		0.585		0.724
Shanghai	1 (Referent)		1 (Referent)	
Guangzhou	1.139 (0.863–1.503)	0.358	1.134 (0.834–1.542)	0.423
Harbin	1.130 (0.851–1.501)	0.397	1.048 (0.762–1.441)	0.775
**Multivariable analysis**				
T stage (T4 vs. T3)	1.151 (0.891–1.487)	0.283	1.399 (1.051–1.861)	0.021
N stage		0.000		0.000
N0	1 (Referent)		1 (Referent)	
N1	2.144 (1.600–2.874)	0.000	2.526 (1.809–3.528)	0.000
N2	2.530 (1.805–3.546)	0.000	2.808 (1.919–4.110)	0.000
Pathology type (adenocarcinoma vs. other)	NA	NA	0.663 (0.423–1.039)	0.073
Differentiation (moderate and well vs. poor)	1.176 (0.883–1.565)	0.268	1.077 (0.784–1.478)	0.648
Lymphatic invasion (positive vs. negative)	0.948 (0.699–1.285)	0.731	1.142 (0.818–1.593)	0.436
Vascular invasion (positive vs. negative)	0.837 (0.611–1.147)	0.269	1.019 (0.736–1.411)	0.911
Postoperative CEA level (positive vs. negative)	3.149 (2.426–4.088)	0.000	3.414 (2.549–4.574)	0.000
CEA increment (yes vs. no)	2.708 (2.106–3.482)	0.000	2.373 (1.783–3.157)	0.000

### Elevated Postoperative CEA and CEA Increment Predicted Worse Prognosis of Stage II and III CRC Patients

**Figure 2:**
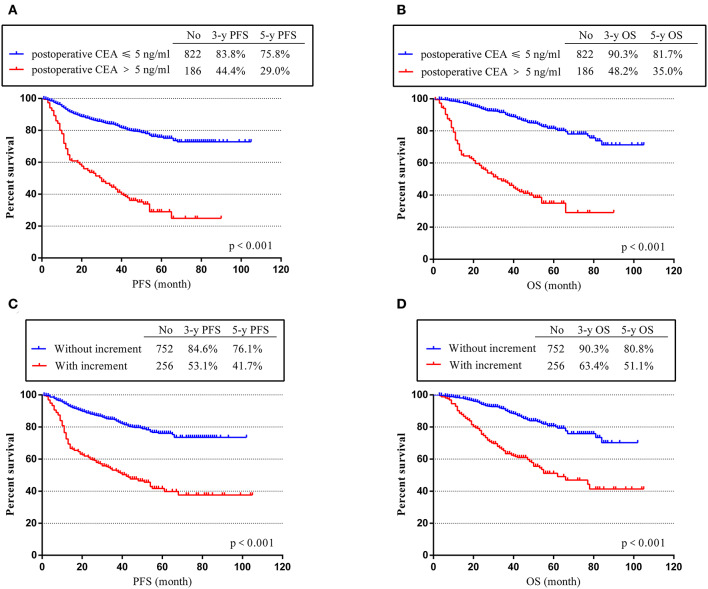
Progression-free survival time (PFS) and overall survival (OS) according to postoperative carcinoembryonic antigen (CEA) level and post-preoperative CEA increment. **(A)** K-M curves of PFS based on postoperative CEA level. **(B)** Kaplan–Meier (K-M) curves of OS based on postoperative CEA level. **(C)** K-M curves of PFS based on CEA increment. **(D)** K-M curves of OS based on CEA increment.

To assess the effects of positive postoperative CEA and CEA increment on the survival time of stage II and III CRC, Kaplan–Meier (K-M) survival curves were used according to our follow-up data. Our results showed that patients with positive postoperative CEA and positive increment had poor PFS and OS. The 3- and 5-year PFS rates for patients with negative postoperative CEA were 83.8 and 75.8%, much higher than patients with positive post-CEA (44.4% and 29.0%, *p* < 0.001) (Figure 2A). Similarly, the 3- and 5-year PFS rates were only 53.1 and 41.7% in patients with CEA increment, which was much worse than patients without increment (84.6 and 76.1%, *p* < 0.001) (Figure 2C). As for OS, we got same conclusions. The 3- and 5-year rates decreased from 90.3 and 81.7% in patients with negative postoperative CEA to 48.2 and 35.0% in patients with positive post-CEA (*p* < 0.001) (Figure 2B). For patients without CEA increment, the 3- and 5-year OS rates were 90.3 and 80.8%, better than patients with CEA increment (63.4 and 51.1%, *p* < 0.001) (Figure 2D).

### PFS and OS Differences Among Four Groups

**Figure 3:**
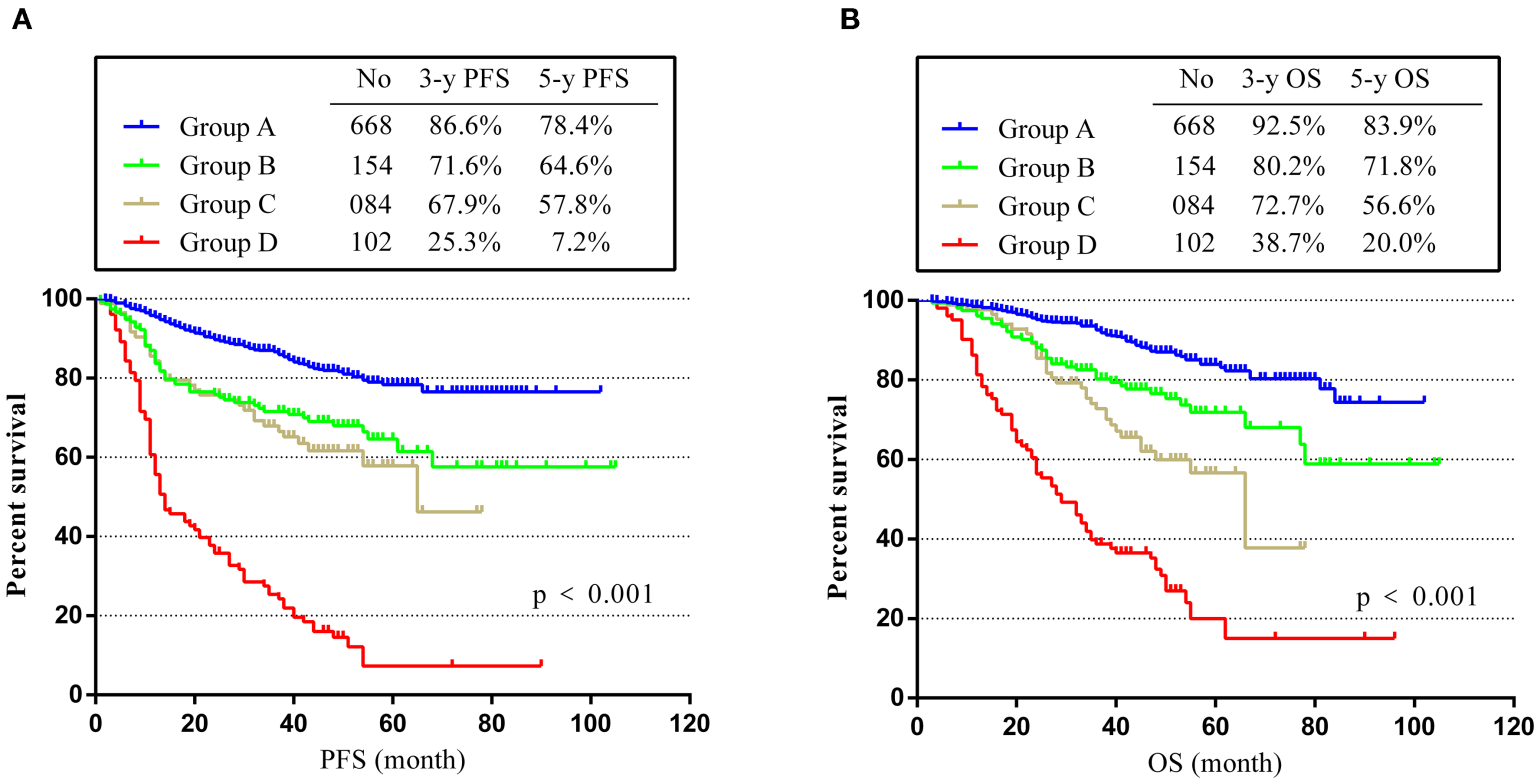
PFS and OS in different groups. **(A)** K-M curves of PFS in different groups. **(B)** K-M curves of OS in different groups.

As described in the Experimental section, we divided the patients into four groups (A, B, C, and D). As shown in Figure 3, group A had the best prognosis, while group D had the worst. The 3- and 5-year PFS rates decreased from 86.6 and 78.4% in group A to 25.3 and 7.2% in group D (*p* < 0.001) (Figure 3A). Consistent with the trend of PFS, the 3- and 5-year OS rates for group A were 92.5 and 83.9%, much higher than rates of group D (only 38.7 and 20.0%, *p* < 0.001) (Figure 3B). These results showed that patients in group D with positive postoperative CEA and CEA increment had the worst prognosis, while patients in group A with normal postoperative CEA and without CEA increment had the highest PFS and OS rates. For groups B and C, we could see that they had similar PFS, but the OS of group C was worse. This phenomenon suggested that elevated postoperative CEA may have more important effects on the prognosis of stage II and III CRC patients.

### Subgroup Analyses to Test the Effect of CEA Levels on Prognosis of CRC

**Figure 4:**
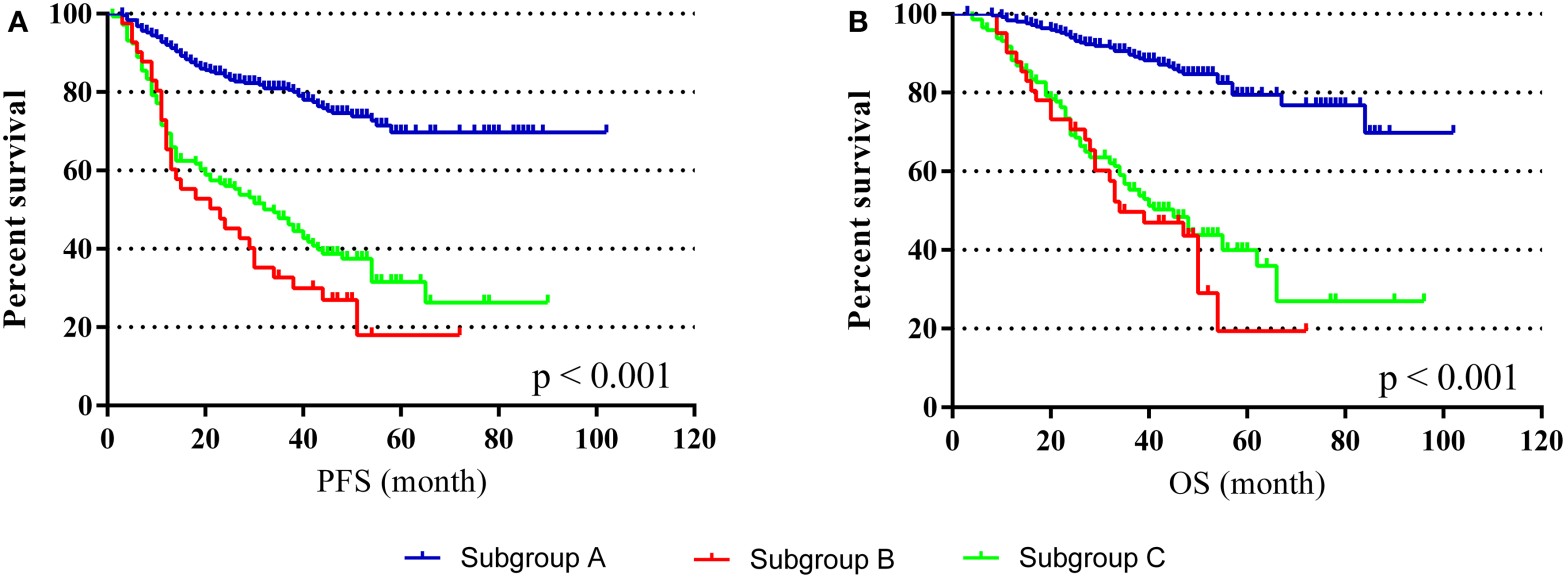
PFS and OS in different subgroups. **(A)** K-M curves of PFS in different subgroups. **(B)** K-M curves of OS in different subgroups.

To investigate the effect of perioperative abnormal CEA on the prognosis of CRC, we performed subgroup analyses. The subgroups were as follows: (Subgroup A) patients with positive preoperative CEA but normal postoperative CEA after radical surgery, *n* = 252; (Subgroup B) patients with normal preoperative CEA but positive postoperative CEA after radical surgery, *n* = 41; (Subgroup C) patients with positive preoperative CEA and positive postoperative CEA after radical surgery, *n* = 145. K-M curves illustrated that sub-B and sub-C had a worse PFS and OS than A, and sub-B was the worst (*p* < 0.001) (Figure 4). By comparing sub-A and sub-C, the results demonstrated that positive postoperative CEA patients had a poor prognosis even after radical resection. Furthermore, the survival of sub-B was worse than that of sub-C, indicating that patients with normal preoperative CEA but positive postoperative CEA had the worst prognosis in these subgroups.

## Discussion

In our study, 1,008 patients with stage II and III CRC were enrolled. Our results suggested that positive postoperative CEA was associated with clinical stage, T stage, N stage, tumor differentiation, lymphatic and vascular invasion, while post-preoperative CEA increment was related to clinical stage, T stage, N stage, tumor differentiation, and lymphatic invasion. Besides, our multivariable analyses demonstrated that positive postoperative CEA and post-preoperative CEA increment were independent prognostic factors for CRC. Patients with elevated CEA level and CEA increment had shorter PFS and OS than patients with normal CEA and without increment. Furthermore, group D patients had the worst prognosis, and positive postoperative CEA had negative impacts on prognosis of CRC. Our subgroup analyses revealed high hazard of recurrence and poor survival in patients with perioperative CEA elevation, consistent with a recent study (18).

Similar to our study, the early postoperative CEA percent drop may be a helpful factor for the prognosis of colon cancer, but the influence of preoperative and postoperative CEA trends on survival has not well been demonstrated (19). Huang et al. conforms that CEA reduction ratio is a prognostic factor in rectal cancer patients who receive chemoradiotherapy and radical surgery (20). Serum CEA alone is less sensitive to detecting CRC recurrence, even though the threshold is low (21). Nevertheless, CT or CEA test each provides a reliable rate of recurrence with minimal follow-up after surgical treatment, and combining CEA and CT shows no advantage (22). Another study suggests that postoperative CEA limit is 15 ng/ml, with a high chance of recurrence after resection for colorectal liver metastasis (11). The same conclusion in a retrospective cohort analysis shows that patients with normal postoperative CEA have 14.9% higher 3-year RFS than patients with elevated post-operative CEA (17). Serum CEA is also correlated with RAS-mutant allele fraction (23).

According to current guidelines, patients undergoing radical surgery for stage II and III CRC need to test serum CEA every 3–6 months (24–27). However, these guidelines do not have individual follow-up and adjuvant therapy advice. Therefore, in clinical practice, should we refer to the levels of perioperative serum CEA when we give patients treatment recommendations? In addition, we also found that the number of preoperative elevated tumor markers also had important impact on the prognosis of CRC, including CEA, CA19-9, CA242, and CA125 (28). We believe that serum tumor markers have great value in CRC, but these markers have not been paid enough attention in the clinic. Thus, our study may provide some references for clinical workers in this field. Admittedly, there are some shortcomings in our research. First, this is a retrospective study, while prospective studies demonstrating the significance of CEA in CRC are more convincing. Second, our study included only one indicator, CEA. Other serum tumor markers were not analyzed. Finally, patients in our study are all Chinese.

In general, the treatment of stage II and III CRC after radical surgery still has some controversial problems (29, 30). Our study demonstrates the effects of postoperative CEA level and CEA increment on the prognosis of stage II and III CRC. Thus, our results will provide useful information for clinical references in the follow-up treatment of CRC patients.

## Conclusion

Positive postoperative CEA and CEA increment are independent prognostic factors for stage II and III CRC. Patients with elevated postoperative CEA level and positive CEA increment have the worst PFS and OS compared to other groups. Our results may be helpful to the adjuvant treatment of stage II and III CRC after radical surgery.

## Data Availability Statement

The datasets generated for this study are available on request to the corresponding author.

## Ethics Statement

The studies involving human participants were reviewed and approved by The ethics committee of Shanghai Jiao Tong University Affiliated Sixth People's Hospital. The patients/participants provided their written informed consent to participate in this study.

## Author Contributions

ZW conceived the project. WY, LY, and NS collected the clinical and follow-up data from Shanghai Jiao Tong University Affiliated Sixth People's Hospital. XW and ZC collected the clinical and follow-up data from the Sixth Affiliated Hospital of Sun Yat-sen University. GW and LX collected the clinical and follow-up data from the Second Affiliated Hospital of Harbin Medical University. WY analyzed all data and wrote the manuscript.

## Conflict of Interest

The authors declare that the research was conducted in the absence of any commercial or financial relationships that could be construed as a potential conflict of interest.
